# Editorial: Neural correlates of visual learning and object representation in inferior temporal lobe

**DOI:** 10.3389/fnbeh.2025.1584584

**Published:** 2025-03-25

**Authors:** Mark A. G. Eldridge, Yasuko Sugase-Miyamoto

**Affiliations:** ^1^Biosciences Institute, Newcastle University, Newcastle upon Tyne, United Kingdom; ^2^Human Informatics and Interaction Research Institute, National Institute of Advanced Industrial Science and Technology (AIST), Tsukuba, Japan

**Keywords:** macaque monkey, ventral visual pathway, inferior temporal cortex, neurons, representation, learning, modeling

The inferior temporal cortex (IT) sits at the apex of the ventral visual stream, a neural pathway specialized in the processing of object identity (Mishkin et al., 1983). IT receives dense anatomical inputs from visual areas V4 and V2 and sends projections to the perirhinal cortex, medial temporal lobe, frontal cortex, and various subcortical regions (Distler et al., 1993; Webster et al., 1991). Neurons in IT tend to have narrow tuning for complex conjunctions of features, and their response properties are generally stable across multiple presentations of the same stimulus (Desimone et al., 1984; Gross et al., 1972). These observations are consistent with the critical role of IT in object perception. However, IT has also been shown to be critical for several forms of visual object memory, and the activity of neurons in this region can be modulated during learning (Fahy et al., 1993; Pearl et al., 2024).

Despite the growing body of literature demonstrating a role for subregions of the inferior temporal cortex in the learning of tasks that place demands on visual memory (Emadi and Esteky, 2014; Fuster and Jervey, 1981; Koida and Komatsu, 2007; Sigala and Logothetis, 2002), relatively little is known about the specific mechanisms via which neuronal activity in these regions subserves the behavioral functions attributed to them. The current Research Topic collection includes articles that explore such mechanisms in the inferior temporal cortex of non-human primates (NHP) using multielectrode array recordings, electrocorticography, and aspiration lesions, along with a review of the responses of IT neurons to visual experience and computational modeling of object recognition in the ventral visual stream.

Shimizu et al. recorded from subregions of IT—areas TE and TEO—using multielectrode arrays, while monkeys learned to categorize images based on the similarity of perceptual features. Neurons in TE encoded category learning more strongly than those in TEO. The time course of the neural responses in TE was consistent with a feedback component from other brain areas.

Ichwansyah et al. collected electrocorticography (ECoG) recordings from IT and dorsomedial prefrontal cortex (dmPFC) while monkeys learned to categorize video clips of animate and inanimate objects. Subregions of both areas carried the information necessary for animacy category decoding, but an interconnected network of subregions within IT demonstrated the highest category selectivity in several frequency bands. Similar to the Shimizu et al. study, Ichwansyah et al. also found evidence that feedback projections to the temporal cortex—to a region in the superior temporal sulcus, in particular—contributed to categorization accuracy.

Li et al. performed aspiration removals of area TE or rhinal cortex and compared the abilities of these two groups to control subjects in a test of rapid categorization based on visual perceptual features. Consistent with Shimizu et al. and several published studies (Matsumoto et al., 2016; Eldridge et al., 2018; Setogawa et al., 2021), Li et al. observed impaired performance in the group with TE removals. In contrast, the group with rhinal removals performed the task as accurately as controls but appeared to make decisions more slowly, implicating the rhinal cortex in the process of rapid categorization, of the type needed when distinguishing predator from prey in the wild.

Yamane presented a comprehensive review of the neural mechanisms observed in IT that have the potential to support visual experience/memory across multiple timescales. Among the changes in neural activity reported, repetition suppression is suggested as a fundamental, task-invariant characteristic of neurons in IT, but similar mechanisms may exist in other (earlier) regions of the visual system.

Quaia and Krauzlis presented a model of recognition memory demonstrating that a V1-like layer can recognize objects with >80% accuracy. Thus, they propose that visual recognition may be a parallel process, wherein earlier regions rapidly detect stimuli in the periphery at low resolution, thereby enabling fine foveal recognition supported by IT. This model provides clues as to how IT might contribute to object/face recognition and is consistent with the conclusions of the four other articles in this collection.

Overall, this collection of studies sheds light on the causal links between neural activity and behavioral outcomes, offering thought-provoking ideas about how IT functions in concert with other brain regions to support visual memory.
